# New genomic resources inform transcriptomic responses to heavy metal toxins in the common Eastern bumble bee *Bombus impatiens*

**DOI:** 10.1186/s12864-024-11040-4

**Published:** 2024-11-19

**Authors:** Amy L. Toth, Christopher D. R. Wyatt, Rick E. Masonbrink, Katherine S. Geist, Ryan Fortune, Sarah B. Scott, Emeline Favreau, Sandra M. Rehan, Seirian Sumner, Mary M. Gardiner, Frances S. Sivakoff

**Affiliations:** 1 Department of Ecology, Evolution, and Organismal Biology, Iowa State University, Ames, IA USA; 2 Department of Plant Pathology, Entomology, and Microbiology, Iowa State University, Ames, IA USA; 3 Centre for Biodiversity and Environment Research, University College, London, UK; 4 Genome Informatics Facility, Iowa State University, Ames, IA USA; 5 Department of Zoology, University of Cambridge, Cambridge, UK; 6 Department of Biology, York University, Toronto, ON Canada; 7 Department of Entomology, The Ohio State University, Columbus, OH USA; 8 Department of Evolution, Ecology, and Organismal Biology, The Ohio State University, Marion, OH USA

**Keywords:** Genome assembly, Genome annotation, Urban toxicology, Bee health, Transcriptomics, Long read sequencing

## Abstract

**Background:**

The common Eastern bumble bee *Bombus impatiens* is native to North America and is the main commercially reared pollinator in the Americas. There has been extensive research on this species related to its social biology, applied pollination, and genetics. The genome of this species was previously sequenced using short-read technology, but recent technological advances provide an opportunity for substantial improvements. This species is common in agricultural and urban environments, and heavy metal contaminants produced by industrial processes can negatively impact it. To begin to identify possible mechanisms underlying responses to these toxins, we used RNA-sequencing to examine how exposure to a cocktail of four heavy metals at field-realistic levels from industrial areas affected *B. impatiens* worker gene expression.

**Results:**

PacBio long-read sequencing resulted in 544x coverage of the genome, and HiC technology was used to map chromatin contacts. Using Juicer and manual curation, the genome was scaffolded into 18 main pseudomolecules, representing a high quality, chromosome-level assembly. The sequenced genome size is 266.6 Mb and BRAKER3 annotation produced 13,938 annotated genes. The genome and annotation show high completeness, with ≥ 96% of conserved Eukaryota and Hymenoptera genes present in both the assembly and annotated genes. RNA sequencing of heavy metal exposed workers revealed 603 brain and 34 fat body differentially expressed genes. In the brain, differentially expressed genes had biological functions related to chaperone activity and protein folding.

**Conclusions:**

Our data represent a large improvement in genomic resources for this important model species—with 10% more genome coverage than previously available, and a high-quality assembly into 18 chromosomes, the expected karyotype for this species. The new gene annotation added 777 new genes. Altered gene expression in response to heavy metal exposure suggests a possible mechanism for how these urban toxins are negatively impacting bee health, specifically by altering protein folding in the brain. Overall, these data are useful as a general high quality genomic resource for this species, and provide insight into mechanisms underlying tissue-specific toxicological responses of bumble bees to heavy metals.

**Supplementary Information:**

The online version contains supplementary material available at 10.1186/s12864-024-11040-4.

## Background

Bumble bees are highly recognizable eusocial insects with a global distribution, possessing an annual lifecycle, medium colony size, and morphological caste differences between queens and workers [1]. They are economically important for crop pollination, including use of industrially reared managed species, the most widely used being *B. impatiens* from North America and *B. terrestris* from Europe [2]. Bumble bees also have important roles in the pollination of native plants and thus deliver important ecosystem services by maintaining wild plant communities [3]. In addition, bumble bees are tractable models for studying fundamental questions in biology, due to their complex social behavior, cognitive abilities, ecosystem services as pollinators, and thermoregulatory capacity [4].

While many bumble bee species populations are declining [5], *Bombus impatiens* is stable and well-adapted to a variety of habitats, nectar sources, and climates, including disturbed environments such as agricultural and urban environments [6]. This species is native to eastern and central North America, with a sizable commercial rearing industry. As the most important managed bumble bee in North America, colonies are frequently deployed for pollination of various crops [7] in greenhouses (e.g. tomatoes) as well as in fields and orchards (e.g. for apple and berry crops). In addition to its role as a beneficial wild and managed species, there is concern over pathogen spillover from commercial *B. impatiens* colonies, as well as the possibility that *B. impatiens* may have invasive potential outside of their native range, including in parts of the United States, Canada, Mexico, and Chile [8]. Thus, this species is of considerable practical and economic importance.

*Bombus impatiens* has also been one of the most widely studied bee species in terms of basic biology, with numerous studies on sociality [9], pollination and foraging [10], cognition [11], disease [12], and responses to environmental stress [13]. There have been many genetic and genomic studies on this species, including transcriptomics of social behavior [9], genetically based responses to disease, pesticides and other stressors [14], and population genetics [15]. Thus, high quality genomic resources for this species are highly valuable to the research community as well as to pollination and conservation practitioners. Currently, an Illumina reference genome exists for *B. impatiens* [16]. At the time of writing, 400 primary articles have cited the originally published *B. impatiens* and *B. terrestris* genomes, which were co-published in 2015. Since then, there have been vast improvements in genome sequencing technology and bioinformatic processing of genomic data, especially with the advent of long read “third generation” sequencing technologies [17]. Thus, despite this species’ importance, we lack a modern genome in line with current standards, even though at the current time, numerous less-studied species have higher quality genomes (e.g [18]). There is a clear need for a new, high-quality genome for this species, and here we fill that need.

In addition to genomic resources, one of the primary ways in which *B. impatiens* has been utilized is as a model organism to understand how insects, and more specifically economically valuable pollinating bees, respond to environmental stress [19]. Given concerns about pollinator declines globally, “workhorse” model species such as *B. impatiens*, whose populations are not declining, can be highly practical for studying general bee stress responses without the danger of negatively affecting imperiled populations [20]. As such, there have been numerous studies on *B. impatiens* response to toxins (e.g. pesticides [21], heat stress [22], nutritional stress [23], and habitat loss [24]. Recently, there has been a rising interest in how bumble bees are affected by urbanization [25] and associated stressors such as land use change, pollution, and industrial toxins [26]. *Bombus impatiens* is widespread in urban environments throughout its native range [6] and is thus commonly exposed to the various stressors associated with urban and industrial environments. Urban *B. impatiens* have been important models to study how bees cope with life in cities, including conservation management and responses to urban associated environmental change [27]. One urban stressor that has recently come to light is exposure to heavy metals, especially in formerly industrial areas [28]. Heavy metals such as lead, zinc, aluminum, arsenic, chromium and cadmium can bioaccumulate in urban *B. impatiens* through foraging on contaminated plants, with acute toxicity in larvae and sublethal impacts on adults [29]. Emerging evidence suggests that urban contaminants can lead to behavioral changes in worker *B. impatiens*, as well as fitness costs. Previous studies have shown heavy metal exposure in bees can lead to deficits in foraging behavior [30, 31], reproduction [32], brood development [33], reduced colony growth [34], energy metabolism [35] and elevated mortality [36, 37]. To date, the underlying mechanisms of these effects are unknown; previous studies on other insects have uncovered heavy metal-induced changes in the expression of detoxification pathways, cytochrome P450s, and numerous metabolic pathways [38, 39], but this remains unexplored in bees. Transcriptomics on heavy metal exposed *B. impatiens* bumble bees (using RNA-sequencing) is a useful starting place for understanding potential molecular mechanisms of bees’ biological responses to urban threats such as heavy metals.

The goal of this report is twofold. First, we provide resources to bolster the study of bumble bee biology and health through the release of a high-quality chromosome level genome assembly and annotation for the model bumble bee *B. impatiens*. To do this, we generated a chromosome-level *de novo* assembly using PacBio long reads and chromatin conformation (Hi-C) sequencing. The combination of long-read sequencing, coupled with chromatin conformation sequencing methods can allow the production of chromosome or near-chromosome level assemblies [17]. Second, we use this genome to inform a transcriptomic study on a modern health threat to bumble bees—exposure to heavy metals. By performing a transcriptomic analysis on brains (to probe behavioral changes) and fat bodies (to probe possible effects on metabolic and detoxification pathways), we generate candidate genes and pathways to understand how heavy metals impact bee behavior and physiology. Overall, these resources represent valuable genomic and transcriptomic information that can help us to better understand bumble bees’ basic biology, pollination services, and responses to global change.

## Methods

### Sample collections and preparation for genome sequencing

On August 30, 2020, ten pupae (not sexed, likely a mix of male and female) were collected from inside of a commercial colony of *B. impatiens* (Koppert Biological Systems, Howell, MI, USA) that had been used for squash pollination at the Horticulture Research Station in Ames, Iowa and placed directly onto dry ice, freeze-killing them instantly, and then were stored at -80 °C. These were then sent on dry ice to Dovetail Genomics, LLC (now Cantata Bio, Scotts Valley, CA, USA) and whole bodies used for DNA extractions and genome sequencing. Extraction for DNA was performed by Dovetail using a Qiagen Kit for PacBio DNA Extraction (cat num: 13362, 19060), following the manufacturer’s recommended protocol. PacBio sequencing library preparation used the SMRTbell Express Template Prep Kit 2.0 (PacBio, Menlo Park, CA, USA), following the manufacturer recommended protocol. The library was bound to polymerase using the Sequel II Binding Kit 2.0 (PacBio) and loaded onto a PacBio Sequel II sequencer with 8 M SMRT cells.

For each Dovetail Omni-C library (for Hi-C sequencing), chromatin was fixed in place with formaldehyde in the nucleus. Fixed chromatin was digested with DNase I and then extracted, chromatin ends were repaired and ligated to a biotinylated bridge adapter followed by proximity ligation of adapter containing ends. After proximity ligation, crosslinks were reversed and the DNA was purified. Purified DNA was treated to remove biotin that was not internal to ligated fragments. Sequencing libraries were generated using New England Biolabs Next Ultra enzymes and Illumina-compatible adapters. Biotin-containing fragments were isolated using streptavidin beads before PCR enrichment of each library. The Omni-C library was sequenced on an Illumina HiSeqX platform.

### Genome assembly and annotation

A *de novo* initial assembly of the PacBio reads was conducted by Dovetail (using wtdbg2) and scaffolding was performed with HiRise, with OmniC library reads with MQ > 50 used as input data [34]. Dovetail checked the genomic Pacbio HiFi reads for contamination using BlobTools [40], and no evidence of significant contamination was found (results reported in Supplemental Fig. 1). PacBio read lengths were visualized using NanoPack [41]. OmniC library sequences were aligned to the draft input assembly using BWA (Li, 2013). The separations of OmniC read pairs mapped within draft scaffolds were analyzed by HiRise to produce a likelihood model for genomic distance between read pairs, and the model was used to identify and break putative misjoins, to score prospective joins, and make joins above a threshold. Additional scaffolding was conducted after receiving the *B. impatiens* initial assembly from Dovetail. HiC reads were extracted from bam files using Picard 2.17.0 [42], which were aligned to scaffolds with BWA 0.7.17 and processed with Juicer 1.5.7, 3D-DNA 180,114, and JuiceBox 1.11 [43]. This produced a final assembly, BIMP_3.0, which has been deposited in NCBI at SUB14541561, PRJNA1124924, SAMN41873696, JBEUIP000000000 as BIMP_3.0.

To compare our final assembly (BIMP_3.0) to other published genome assemblies, we obtained assembly statistics with QUAST [44], BUSCO [45], and mapped assemblies to each other with a liftover approach using flo [46]. Specifically, we compared BIMP_3.0 to the most recent previously published assembly for this species (BIMP_2.2 [16, 47]), as well the current ‘gold standard’ of social insect genomes, for the honey bee *Apis mellifera*, Amel_HAv3.1 [48]. To explore synteny between the new (BIMP_3.0) and the most recent previous assembly BIMP_2.2 (GCF_000188095.3), another well assembled model bumble bee species *Bombus terrestris* (iyBomTerr1.2 [49]), as well as to *Apis mellifera* [GCF_003254395.2], we used the jcvi toolkit [50], which uses MCScanX [51] to calculate syntenic regions of the genome. First, we used gffread [52], to extract the gene sequences (gffread -w $sample\.nucl.fa -g genome.fa $sample\.gff_for_jvci.gff3) from the genome and annotation files (gff3). Second, we ran the “jcvi.formats” function in JCVI, to get the genome in .cds format, and the annotation in .bed format. Third, we ran the main JCVI commands to create anchor files which show the corresponding syntenic blocks between two species. With this finally, we could construct the ribbon figure for the three species, by constructing a layout and seqids text file, specifying which species to plot and the appropriate anchor file. All synteny analysis code is available at https://github.com/ISUgenomics/2024_Toth_Bimpatiens.

To annotate *B. impatiens* genes, repeats were identified in the genome using Repeatmodeler 2.0.2 [43] and softmasked with Repeatmasker 4.1.2-p1 [53]. Previously published RNA-seq reads from 115 samples from *B. impatiens* (NCBI Accession numbers SRR15927739-SRR15927792, queens from lab reared colonies [54]), SRR26132658-SRR26132679 (NCBI BioProject PRJNA1019664, male/female gonads and brain), SRR19756248-SRR19756300, and SRR19756302- SRR19756304 (hindgut/gut metatranscriptome [55]) were aligned to the genome using STAR 2.7.6a [56]. These datasets were chosen as RNA evidence for the annotation pipeline over our own RNA-seq data (described below) and other published datasets because they had the longest read length we could find among publicly available *B. impatiens* RNA-sequencing data, and were derived from the brain, which is a tissue with a high diversity of different expressed transcripts. Using Augustus 3.4 [57] models trained with BUSCO 5.12 on hymenoptera_odb10 and by using an extrinsic file to increase scoring on hints matching RNAseq data for introns, exons, and CDS. These Augustus inputs were used by BRAKER 3.0.2 [58] and GeneMark 4.69 [59] to create a gene annotation. Subsequent to this *de novo* annotation, we improved gene models using Genomethreader [60] alignments of proteins from the NCBI *Bombus impatiens* Annotation Release 103. The genes were functionally annotated by querying predicted proteins with Diamond 2.0.25 [61] to the NCBI NR database (downloaded April 2024) and Swiss Prot database (downloaded April 2024). The annotation file (gff format) and functional annotation have been publicly posted in Supplemental Dataset 1.

### Sample collections and preparation for RNA-sequencing

To explore how heavy metal exposure affects gene expression, we utilized samples from a previous study that found colonies fed a cocktail of four heavy metals had a significantly higher brood mortality than controls [33]. The methods were the same as the published study. Briefly, commercial *B. impatiens* colonies (Koppert Biological Systems, Howell, MI, USA) containing one queen and approximately 50 workers were fed a cocktail of heavy metals (arsenic, cadmium, chromium, and lead) for 30 days and compared to control colonies that were not fed heavy metals. This cocktail of heavy metals was chosen because they are commonly found in urban environments and are known to have sublethal effects on adult worker bees [34]. Concentrations were based on levels of heavy metals previously found in bee provisions in a previous study in this area (Cleveland, OH) and were as follows: arsenic (arsenic (III) oxide, As2O3; 0.894 ppm), cadmium (cadmium chloride, CdCl2; 0.276 ppm), chromium (chromium (VI) oxide, CrO3; 0.245 ppm), and lead (lead nitrate, Pb(NO3)2 ; 0.265 ppm). These were added to sucrose solution (50% w/v), and control nectar used an equal quantity of added deionized water instead of heavy metals. Colonies were provided with *ad lib* nectar feeders, replenished daily, and ground honey bee-collected pollen. Bees were allowed to freely forage in outdoor enclosures for 30 days, a time period long enough that eggs laid by the queen would complete their life cycle while exposed to the heavy metal cocktail. On day 28, approximately 10 worker bees from each of the treatment (*n* = 4) and control (*n* = 2) colonies were collected from around the nectar feeders and immediately placed onto dry ice and stored at -80℃ until RNA extraction. Workers at feeders were collected to ensure they had consumed the metal-spiked nectar within the last 4 h, and collecting workers active outside their boxes also prevented disruption to colonies.

Worker heads were removed from storage at -80 °C, then lyophilized at -80 °C at 0.35 Torr for one hour (allowing us to cleanly dissect brains while still frozen) and then dissected on dry ice to preserve RNA integrity. Fat bodies were dissected by thawing abdomens in RNAlater solution, abdomens were opened, and organs removed, leaving the exoskeleton with fat body adhered. All dissection tools were cleaned with 70% ethanol followed by RNase AWAY spray. These dissections resulted in 60 individual bees’ brain samples (*n* = 24 control and *n* = 36 metal exposed) and 67 individual bees’ fat body samples (*n* = 27 control and *n* = 40 metal exposed). Most of the same individual bees were used for RNA-sequencing of both tissues, but there was some non-overlap due to low quality/quantity RNA yields from specific samples that were subsequently excluded from analysis (complete sample details can be found in Supplemental Tables 5 and 6).

For each individual brain or fat body, RNA was extracted using a Qiagen RNEasy Mini Kit (following manufacturer’s instructions) with a DNase treatment. RNA was quantified and quality checked using an Agilent Bioanalyzer. Quant-Seq mRNA libraries were prepared by the Iowa State University DNA Facility following manufacturer’s protocols, and subjected to Illumina Quant-Seq using a HiSEQ 3000 Sequencer. For brain tissues, 50-base single end reads were generated and for fat body tissues, 100-base single end reads were generated. RNA data raw reads have been deposited in NCBI Short Read Archive at PRJNA1124924 GEO: GSE272415, SRX25355904- SRX25356002.

### Bioinformatic analysis of RNA-seq data

RNA sequencing resulted in 305.7 M total reads (average 5.1 M reads per sample) for brains and 382.2 M total reads (average 6.2 M reads per sample) for fat bodies. Alignment rates to the BIMP_3.0 annotation averaged 74.8% for brain samples and 75.0% for fat body. Total read counts and mapping rates, as well as other quality statistics for all samples are provided as multiQC [62]reports in Supplemental datasets 3 and 4. All 60 brain samples and 67 fat body samples passed quality filtering and were used for subsequent analysis. Each RNA-seq dataset was then run through nf-core [63] rnaseq v3.14.0 (using Nextflow version 24.04.2 [64]. Reads were quality filtered using Trimgalore v3.4 [65] and mapped to the BIMP_3.0 gene annotation using STAR alignment mapper [56]. We counted aligned reads using Salmon v1.10.1 and determined differentially expressed genes with DESeq2 [66] and considered results for both an FDR-adjusted *p* < 0.1 and *p* < 0.05. Relationships between mean expression, fold change, and statistical significance were visually explored with volcano and MA plots (Supplemental Figures S3, S4, S5, and S6). Principal components analysis (PCA) was performed using the ‘prcomp’ function in R v4.4.0. The PCA used only the top 500 most variable genes for each tissue, and all components were considered. Gene Ontology analysis was performed on the RNA-seq data for brain. For fat body, there were too few differentially expressed genes, resulting in no significant GO enrichment for any terms, thus GO enrichment results are not reported for fat body. GO analysis was performed using topGO [67], focusing on the 137 genes with a > 0.5 log2 fold change cutoff and < 0.05 p-value cutoff, using a background of all genes that were expressed in at least one sample and using a Bonferroni correction for p-values. The GO database of BIMP_3.0 genes to GO terms, was created by using the Eco-Flow excon pipeline [68]. Briefly, this pipeline uses gffread [52] to derive protein sequences for each gene, then uses Orthofinder [69] to find orthologous groups of proteins with known GO annotations for selected species, and then uses the related GO terms to functionally annotate our BIMP_3.0 genes. We used default settings and sourced GO terms from 7 background species (*Anopheles gambiae*,* Drosophila melanogaster*,* Bombyx mori*,* Apis mellifera*,* Tribolium castaneum*,* Acyrthosiphon pisum*,* Nasonia vitripennis*) and merged their annotations into BIMP_3.0 (https://github.com/ISUgenomics/2024_Toth_Bimpatiens/ has code and additional details on the RNA-seq analysis).

## Results

### Genome assembly

**Fig. 1:**
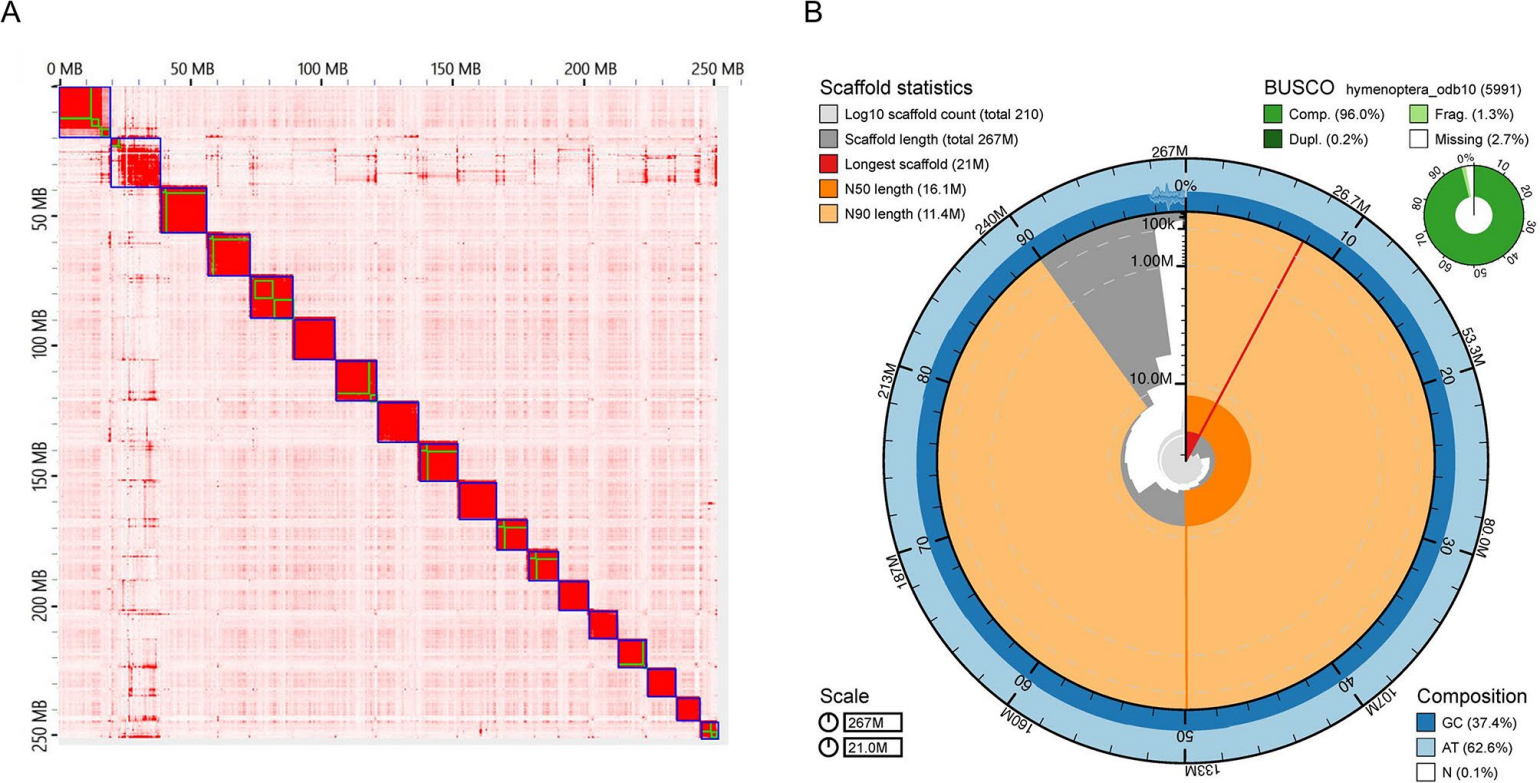
**(A)** HiC contact maps from the genome assembly resulting from the final manual curation with Juicer. The x axis represents the read position of the first read of the read pair, and the y axis represents the read position of its mate, and colored boxes represent distinct scaffolds, with blue squares added to delimit individual chromosomes. The genome includes 18 scaffolds in the final assembly (corresponding to the known 18 chromosomes found in *B. impatiens*). (**B)** BlobTools Snail Plot of the re-scaffolded assembly showing length distribution of scaffolds, BUSCO scores of the final assembly, and GC/AT/N composition

The PacBio sequencing resulted in 5.47 M raw reads and 145.1 Gb of total sequence; the read distribution indicates long, intact DNA with an N50 read length of 38,124 bp (Supplemental Figure S2). This is estimated to represent 544x coverage of the genome of *B. impatiens* (based on total sequence amount divided by final assembly size). An initial assembly using HiRise resulted in 786 scaffolds, with a total assembled genome size of 266.30 Mb and an N50 scaffold length of 15.71 Mb (Supplemental Table S1). Further manual curation of the HiRise assembly using Juicer resulted in several improvements, reducing the number of scaffolds to 210, with a total assembled genome size of 266.6 Mb, which represents a larger estimated genome size than the previously published genome size of 247 Mb [16]. Compared to the initial assembly, the Juicer enabled scaffolding of 6% more contigs to the pseudomolecules, and in the final assembly 100% of scaffolds were > 1Kb in length. The L90 scaffold count (i.e. set of the smallest number of scaffolds for which the added length covers 90% of the estimated genome length) for the initial assembly was 19, and this went down to 16 for the final Juicer assembly. The initial assembly produced 20 predicted chromosomes. For the final assembly BIMP_3.0, most of the assembly (97.80%) was placed into scaffolded contigs, and a HiC contact map of the final assembly (Fig. 1A) shows 18 predicted chromosomes. The known karyotype of this species is 18 chromosomes [70], thus our final assembly likely represents a fairly complete chromosome level assembly for this species.

The GC content of the BIMP_3.0 genome assembly is 37.39% (Fig. 1B), which is similar to other sequenced bumble bee genomes [18], and very similar to previous estimates for this species from its prior genome assembly BIMP_2.2 [16]. We assessed genome completeness using BUSCO, with results from the initial Dovetail and final assemblies shown in Supplemental Table S2. Overall, these scores were high, especially for the final BIMP_3.0 assembly, which had 97.6% of highly conserved Eukaryote genes present in the assembly (255 out of 269 total BUSCO groups searched) and 96.0% of highly conserved Hymenoptera genes (5752 out of 5991 total BUSCO groups searched).

### Comparison to other genome assemblies

**Fig. 2:**
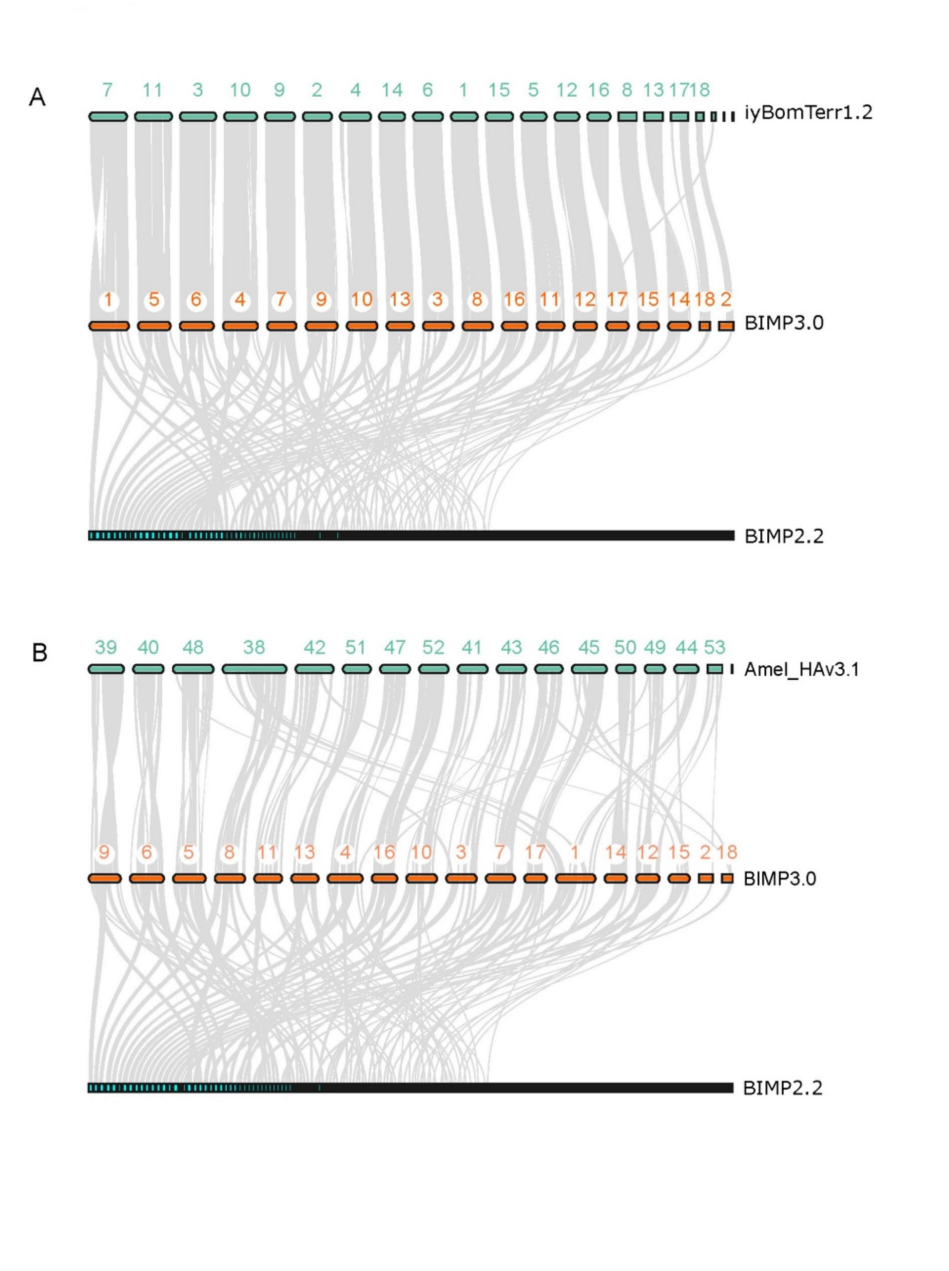
Synteny comparison of the previous BIMP_2.2 genome assembly with the new BIMP_3.0 assembly, alongside modern assemblies from two model bee species, *Bombus terrestris* (**A**) and *Apis mellifera* (**B**). BIMP_3.0 is a highly contiguous assembly with large syntenic blocks with both model bee species, whereas the previous BIMP_2.2 assembly is much more fragmented. Chromosomes in *Apis* are labeled with the last two digits of the chromosome, all starting with 376##

Compared to the most recent prior release of the *B. impatiens* assembly BIMP_2.2, which was based on short read sequencing, as well as the “gold standard” *Apis mellifera* genome (based on long read sequencing), we see overall similar genome statistics (Table 1; Fig. 1B) in terms of GC content, total genome size, BUSCO scores. BIMP 2.2 was a high-quality assembly from 3 different Illumina sequencing projects [16]. However, likely due to our use of long-read sequencing and Hi-C mapping, the BIMP_3.0 assembly covers more than previous high-quality Illumina assembly BIMP_2.2. For example, a liftover mapping approach comparing BIMP_3.0 to BIMP_2.2 resulted in 97.1% coverage, whereas mapping BIMP_2.2 to BIMP_3.0 resulted in 87.8% coverage. This suggests our new assembly gained nearly 10% of the genome missing from BIMP_2.2. We also produced a large improvement in the contiguity of the BIMP_3.0 assembly compared to the previously published assembly, with a large reduction from 2,506 contigs in BIMP_2.2 down to 210 contigs in BIMP_3.0. Also, the largest contig in BIMP_2.2 was only 5.5 Mb, compared to 21.0 Mb in the current assembly. The new BIMP_3.0 assembly also includes more repetitive DNA (21.21% compared to 17.9% in BIMP_2.2), likely due long-reads allowing for improved ability to resolve the assembly in repetitive regions. Overall, the new BIMP_3.0 genome assembly is close to the “gold standard” honey bee genome [48] in terms of the number of contigs, longest contig, and completeness (Table 1). In addition, our synteny analysis revealed large syntenic blocks between both *B. terrestris* and *Apis mellifera* chromosomes and our newly generated BIMP_3.0 putative chromosomes, whereas the previous assembly BIMP_2.2 is highly fragmented, and scaffolds were scattered across multiple *A. mellifera* chromosomes. (Fig. 2).

**Table 1:** Genome assembly statistics and annotation counts for the new BIMP_3.0 genome compared to the prior short read-based genome for this species (most recent assembly BIMP_2.2 [47]), as well as the long read genome of the model bee species *A. mellifera* [48]. The annotation version referred to for *Bombus impatiens* is NCBI Release 103, and for *A. Mellifera* NCBI Release 104

Assembly statistics	B. impatiens BIMP_2.2	B. impatiens BIMP_3.0	A. mellifera Amel_HAv3.1
Total aligned length (Mbp)	237.94	238.49	223.84
GC %	0.3776	0.3744	0.3253
Contigs	2,506	210	227
Largest contig (Mbp)	5.5	21	13.4
Scaffold L50	54	50	13
Repeats %	0.179	0.212	0.079
Complete Single Copy BUSCO Hymenoptera %	98.3%	96.0%	98.3%
**Annotated Features**			
Total Genes	13,161	13,938	12,332

### Genome annotation

The annotation using BRAKER3 resulted in 13,938 annotated genes with 35,653 mRNA transcripts. Because we used multiple gene prediction strategies and integrated *de novo* and homology-based predictions, multiple transcript models were produced for each gene to enable the user to identify the most realistic transcript. Due to this strategy, we annotated 11,182 more transcripts than the previous annotation (BIMP_2.2, NCBI Release 103). Average gene length was 8,561 bp, with a range of 115 bp to 435,723 bp (Supplemental Table S3). We assessed the completeness of the annotation using BUSCOs, revealing 98.8% complete Eukaryote BUSCO genes and 95.7% complete Hymenoptera genes (Supplemental Table S2).

Compared to the prior BIMP_2.2 gene annotation (NCBI release 103), our annotation added 777 new annotated genes to the genome of *B. impatiens.* To facilitate comparisons with the large number of prior studies using BIMP_2.2, we used Genomethreader 1.7.1 alignments of the BIMP_2.2 predicted proteins to cluster our de-novo transcripts with this published dataset to better allocate similar transcripts into a single gene. We found that 91.9% of these proteins aligned (22,492 out of 24,471) and used this information to provide a conversion list between the old BIMP_2.2 and the new BIMP_3.0 annotation (Supplemental Dataset 2). We functionally annotated protein coding genes via Diamond queries to NCBI NR and Swiss-Prot databases resulting in a functional annotation for 93.7% of the 35,653 proteins matching to *B. impatiens* mRNAs, an 11.8% increase over BIMP_2.2. Among these, 204 actively transcribed transposon genes were identified in the genome, along with over 19,000 retroelements (summary of transposable element content in Supplemental Table S4). Functional annotations have been made available on our repository; link provided in Supplemental Dataset 1.

***RNA-sequencing of Heavy Metal Exposed Bees***

**Fig. 3:**
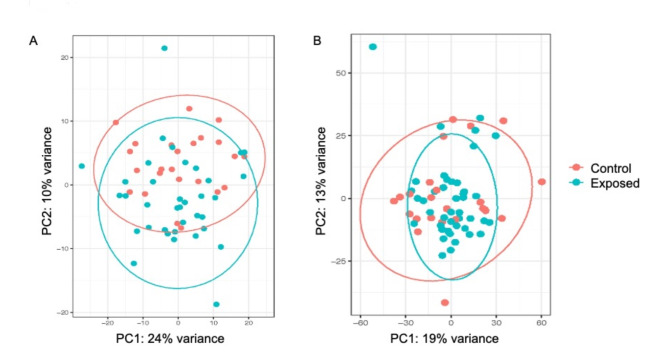
Principal component plots showing principal components (PC) 1 and 2 from overall gene expression patterns, where each dot represents an individual sample from either control or heavy metal exposed worker *B. impatiens.* (**A**) Brain tissue (**B**) fat body tissue

Overall, there was a large amount of overlap in global gene expression patterns between control and heavy metal exposed bees. A principal components analysis of the top 500 most variable transcripts for each tissue revealed more separation between samples in brain tissue compared to fat body tissue (Fig. 3). For brain, the first two PCs explained 34% of the variation in gene expression, and for fat body, the first two PCs explained 32% of the gene expression variation. DESeq2 based differential expression analysis, using an adjusted p-value significance threshold of *p* < 0.05, resulted in 603 differentially expressed transcripts found in brains in response to heavy metal exposure, which amounts to 4.6% of analyzed transcripts. Of those, slightly more than half were upregulated (319 transcripts) compared to downregulated (284 transcripts). There was little differential expression in the fat body, with only 0.23% of transcripts showing evidence of differential expression (34 differentially expressed, with 23 upregulated and 11 downregulated) at adjusted *p* < 0.05 (Supplemental Table S7). There was little overlap in which genes were differentially expressed in the two tissues; the only two genes that overlapped were BimpGene_00013159 (*protein lethal(2)essential for life*) and BimpGene_00007967 (*serine/arginine-rich splicing factor 7*).

**Fig. 4:**
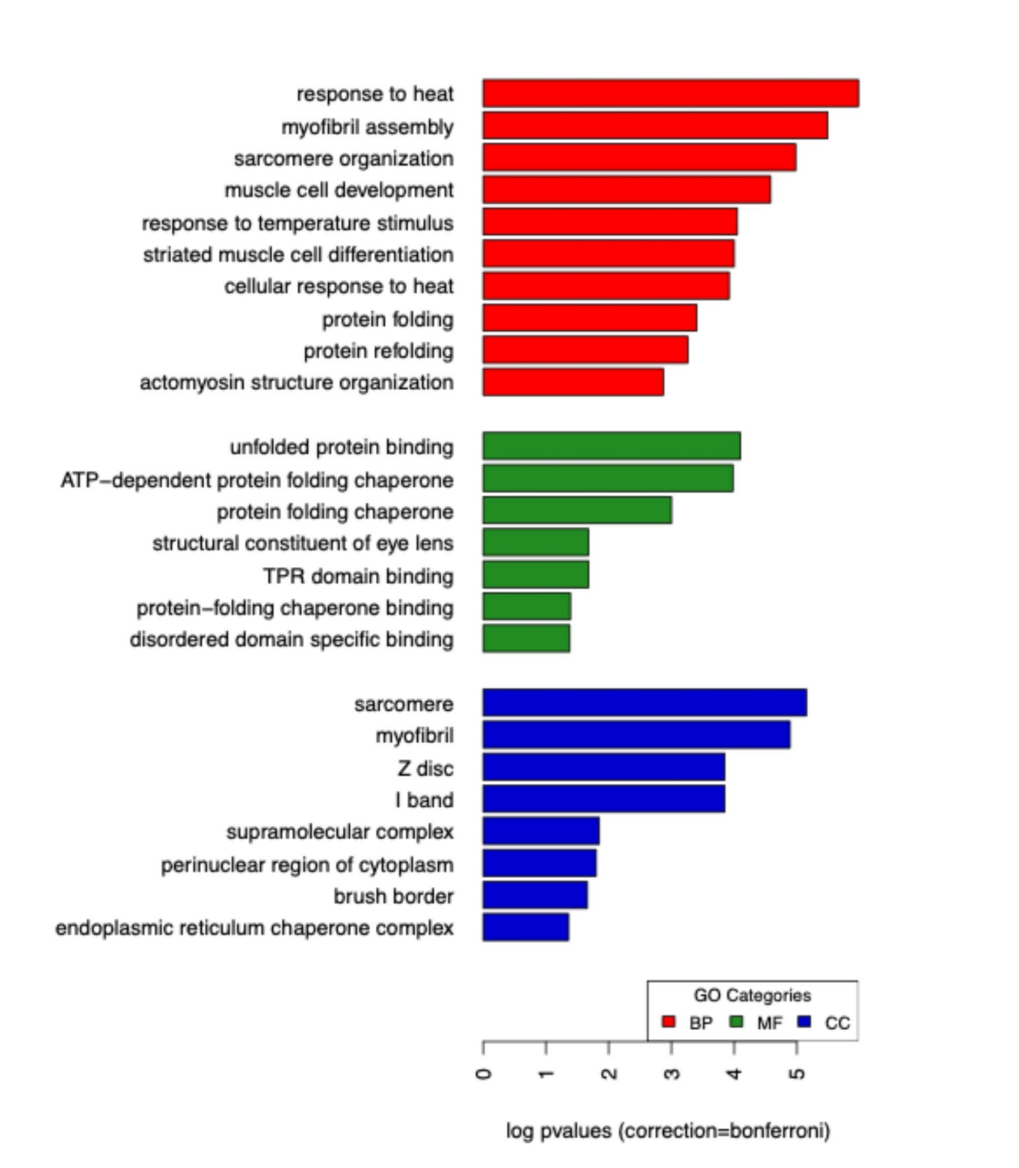
Gene ontology analysis results showing biological process (BP), molecular function (MF), and cellular compartment (CC) for *B. impatiens* brains differentially expressed in response to heavy metal exposure

Similar overall patterns (but with higher numbers of differentially expressed transcripts) were found with a more relaxed p-value threshold of *p* < 0.10 (Supplemental Table S7). A full list of differentially expressed genes, along with their associated GO terms and putative gene names (best BLAST hits) is provided in Supplemental Dataset 1. GO terms associated with brain differential expression included biological processes such as response to heat, temperature, protein folding and refolding, as well as several processes related to muscle function (e.g. muscle development and muscle cell differentiation, myosin and myofibril function) (Fig. 4). Associated molecular functions of differentially expressed genes include several related to protein folding and chaperone functions, suggesting metal exposure could disrupt protein folding in the brain. GO enrichment analysis was not possible on the fat body RNA-seq data because of the small number of differentially expressed genes, but interesting individual gene candidates for fat body responses to heavy metal exposure include *protein lethal(2)essential for life* (a heat shock protein), two major structural proteins (*collagen alpha-1(I) chain*,* pupal cuticle protein-like*), a homeotic developmental gene (*homeotic protein empty spiracles-like*), an organ morphogenesis gene (*bric-a-brac 2-like*), among several others. A list of top differentially expressed fat body genes (adjusted p-value < 0.05 and > 2 fold change difference) is provided in Table 2.

**Table 2:** Examples of differentially expressed genes (adjusted *p* < 0.05, fold change > 2) in fat bodies of bees exposed to heavy metals. Note that there were also two uncharacterized proteins upregulated and one downregulated (not shown in table but BIMP_3.0 gene identifiers available in Supplemental dataset 1)

Top upregulated genes (*P* < 0.05, FC > 2)			
BIMP_3.0 Gene Identifier	Putative gene name	Best hit from	Function
BimpGene_00005435	*trypsin Blo t 3*	*Bombus impatiens*	digestive enzyme
BimpGene_00013019	*titin*	*Apis florea*	molecular spring muscle protein
BimpGene_00006031	*G protein-coupled receptor kinase 5 isoform X2*	*Bombus impatiens*	serine/threonine protein kinase
BimpGene_00005568	*homeotic protein empty spiracles-like*	*Bombus bifarius*	developmental control of antennal and mandibular segment identity
BimpGene_00010558	*flocculation protein FLO11-like*	*Megachile rotundata*	adhesive cell-cell interactions
BimpGene_00009799	*dynein heavy chain 7*	*Bombus bifarius*	motor proteins that aid sliding between ciliary doublet microtubules
BimpGene_00008936	*serine protease hepsin*	*Cephus cinctus*	regulation of glucose, lipid, and protein metabolism.
BimpGene_00001758	*collagen alpha-1(I) chain*	*Bombus impatiens*	major structural protein
BimpGene_00013691	*N-acetylglucosaminyl-phosphatidylinositol de-N-acetylase*	*Bombus impatiens*	enzyme from the GPI biosynthetic pathway
BimpGene_00013159	*protein lethal(2)essential for life*	*Bombus impatiens*	heat shock protein
**Top downregulated genes** (***P* < 0.05, FC > 2)**			
**BIMP_3.0 Gene Identifier**	**Putative gene name**	**Best hit from**	**Function**
BimpGene_00006095	*pupal cuticle protein-like*	*Bombus vosnesenskii*	major structural protein of the cuticle
BimpGene_00000672	*SCY1-like protein 2 isoform X1*	*Bombus impatiens*	clathrin-dependent trafficking at plasma membrane
BimpGene_00002104	*feline leukemia virus subgroup C receptor-related protein 2-like isoform X1*	*Bombus bifarius*	virus receptor
BimpGene_00000729	*bric-a-brac 2-like*	*Bombus impatiens*	organ morphogenesis and regulation of stem cell differentiation
BimpGene_00007889	*ras-like protein family member 10B*	*Eufriesea mexicana*	enable G protein activity and GTP binding activity

## Discussion

This article presents high quality genomic resources, specifically a chromosome level genome assembly and annotation, for the model bumble bee *Bombus impatiens*. Despite its importance as a commercially reared species, ecologically important native pollinator, and workhorse for the study of bumble bee biology, researchers have been relying for several years on an older version of the genome based on short-read sequencing technology [16]. While the resource has been highly valuable and utilized in 400 studies, the assembly was highly fragmented, thus preventing the annotation of more optimal gene models. The BIMP_3.0 assembly and annotation provided here provide vast improvements, with an estimated 10% more of the genome covered by our new assembly. Using long read PacBio sequencing and HiC chromatin mapping, along with manual curation using Juicer, we produced an assembly with 18 putative chromosomes. This number reflects the known karyotype of this species [70] and shows high synteny to the 18 chromosomes of many other bee species, including the current gold standard genome assembly of the honey bee *Apis mellifera*. In addition, our assembly shows high completeness, with over 96% of conserved Eukaryotic BUSCO genes detected in the assembly.

Our new BRAKER3-based annotation BIMP_3.0 of the *B. impatiens* genome utilized evidence from the prior BIMP_2.2BIMP_2.2 annotation (from NCBI) as well as RNA-sequencing data [54], resulting in 13,938 predicted genes. This represents over 700 new gene models for this species, which can be of utility in many applications, from transcriptomics to population genomics to studies of molecular evolution. The annotations identified a large number of putative transposable elements (over 19,000 retroelements). Because many studies have utilized previous annotations including most recently, BIMP_2.2, we have provided a conversion list of matching gene models, so that comparative analyses between studies utilizing the old annotation and new studies using the current annotation can be compared and co-analyzed. All raw reads, assembly, gene models, functional annotations, and a gene conversion list have been made publicly available via NCBI and GitHub for use by the scientific community.

This study also provides the first transcriptomic data examining bumble bee responses to a commonly encountered source of urban stress in bees, exposure to heavy metals. Bumble bees and other pollinators may encounter heavy metals from industrial activity in urban areas, including ingesting heavy metals in nectar and pollen from flowers growing in contaminated areas [28]. Recent studies have demonstrated the potential for bumble bees to bioaccumulate heavy metals, and this can result in numerous impacts on bumble bee colonies, including lower brood survival [33], reduced colony growth [34], and altered foraging efficiency [30, 31]. A companion study to the current study [33], using the same bees analyzed in the current study found that field-realistic levels of exposure of *B. impatiens* through contaminated nectar sources results caused elevated brood mortality, and prior studies showed the potential for sublethal behavioral effects on workers, specifically reduced visit duration to flowers [30, 31]. To begin to investigate possible molecular mechanisms by which heavy metals may alter worker bee physiology and behavior, we examined the transcriptomic responses in individual bees from semi-field reared colonies exposed to either a cocktail of heavy metals or control (no metal exposure) and analyzed two tissues, the brain and the fat body. We found significant transcriptomic responses to heavy metal exposure in both tissues. In the brain, we found a larger transcriptomic response than in the fat body, with over 600 differentially expressed genes, representing 4.6% of annotated genes, in the brain altered by heavy metal exposure. Brain differential expression included several genes with functions related to heat shock stress, protein folding, and muscle function. These data suggest heavy metals may in some way mimic heat stress responses and potentially lead to protein misfolding; thus, they could potentially alter neuronal function through perturbations of protein folding in brain tissue. Previous research in other organisms has also identified heavy metals as causing protein misfolding and aggregation [71, 72], thus our results suggest one potential mechanism by which bee brain function (and thus foraging performance) may be altered by heavy metal exposure. It is not clear why we also detected a strong signal for muscle cell development, differentiation, and myosin/myofibril function, as muscle tissue should have been removed during brain dissections. We speculate this is either related to (1) small amounts of muscle tissue contamination in brain dissections, or (2) pleiotropic roles of muscle development/differentiation genes in some aspects of neural function such as small molecule transport or ionic balance.

In contrast, we found fewer differentially expressed genes in the fat body. The fat body is one of the main tissues involved in detoxification processes, and has key functions in energy storage and immunity [73]. The fact that we found relatively few differences in gene expression in the fat body could be attributed to relatively low levels of heavy metal exposure; however, the purpose of our experimental design was to provide field-realistic exposure levels, thus the differences we found, while subtle, are likely to be biologically relevant. The lack of detoxification processes in the fat body may also suggest the fat body does not mount an active detoxification response to heavy metals, at least at the relatively low concentrations used in this experiment. However, the there do apper to be some small effects of heavy metal exposure on fat body gene expression, including some genes with structural and developmental functions. These perturbations could possibly contribute to reduced health, worker efficiency, and lower colony growth in response to heavy metals. Future studies examining specific metals (as opposed to the cocktail used here), or in vitro studies of bee cellular responses to heavy metals, may help to better understand how metals may disrupt key functions such as protein folding in the brain, and basic structural and developmental processes in the fat body.

## CONCLUSIONS

This study provides a chromosome level assembly and annotation for the key model bee species *Bombus impatiens.* This genomic information can be highly useful for numerous future applications, including breeding, population genetics, molecular evolution, transcriptomics, and studies of gene function. In addition, we demonstrate for the first time that field-relevant levels of exposure to urban toxins (heavy metals) have the potential to alter brain function *via* changing the expression of key genes related to stress responses and protein folding. Our data also suggest very little fat body transcriptomic response to heavy metal exposure, suggesting fat bodies of adult worker bees may have limited capacity for heavy metal detoxification. These results provide candidate mechanisms for further investigation into how urban toxins can negatively impact bee health.

## Electronic supplementary material

Below is the link to the electronic supplementary material.

Supplementary Material 1

## Data Availability

The genomic data for BIMP_3.0 have been deposited at DDBJ/ENA/GenBank under the submission number SUB14541561, project number PRJNA1124924, biosample identifier SAMN41873696, and genome code JBEUIP000000000. Transcriptomic data have been deposited in NCBI Short Read Archive at project number PRJNA1124924, GEO: [57], short read datasets SRX25355904- SRX25356002. Gene annotations have been released, and all scripts used to analyze data can be found on GitHub at https://github.com/ISUgenomics/2024_Toth_Bimpatiens.

## Abbreviations

BUSCO: Benchmarking Universal Single-Copy Orthologs
GO: Gene Ontology
NCBI: National Center for Biotechnology Information
PacBio: Pacific Biosystems
SMRT: Single Molecule Real Time
