# Supplementary material for: New genomic resources inform transcriptomic responses to heavy metal toxins in the common Eastern bumble bee Bombus impatiens
Source: BMC Genomics. 2024 Nov 19;25:1106. doi: 10.1186/s12864-024-11040-4 (PMC11575022; doi:10.1186/s12864-024-11040-4)
Supplement: Supplementary file 1 — Supplementary Material 1 [file 12864_2024_11040_MOESM1_ESM.docx]

**SUPPLEMENT**

**Supplemental Figure 1**

BlobTools plots showing GC content of sequencing reads from Dovetail’s PacBio sequencing reads of *B. impatiens* DNA. These plots demonstrate low contamination of samples from non-Arthropod sources of DNA.

**
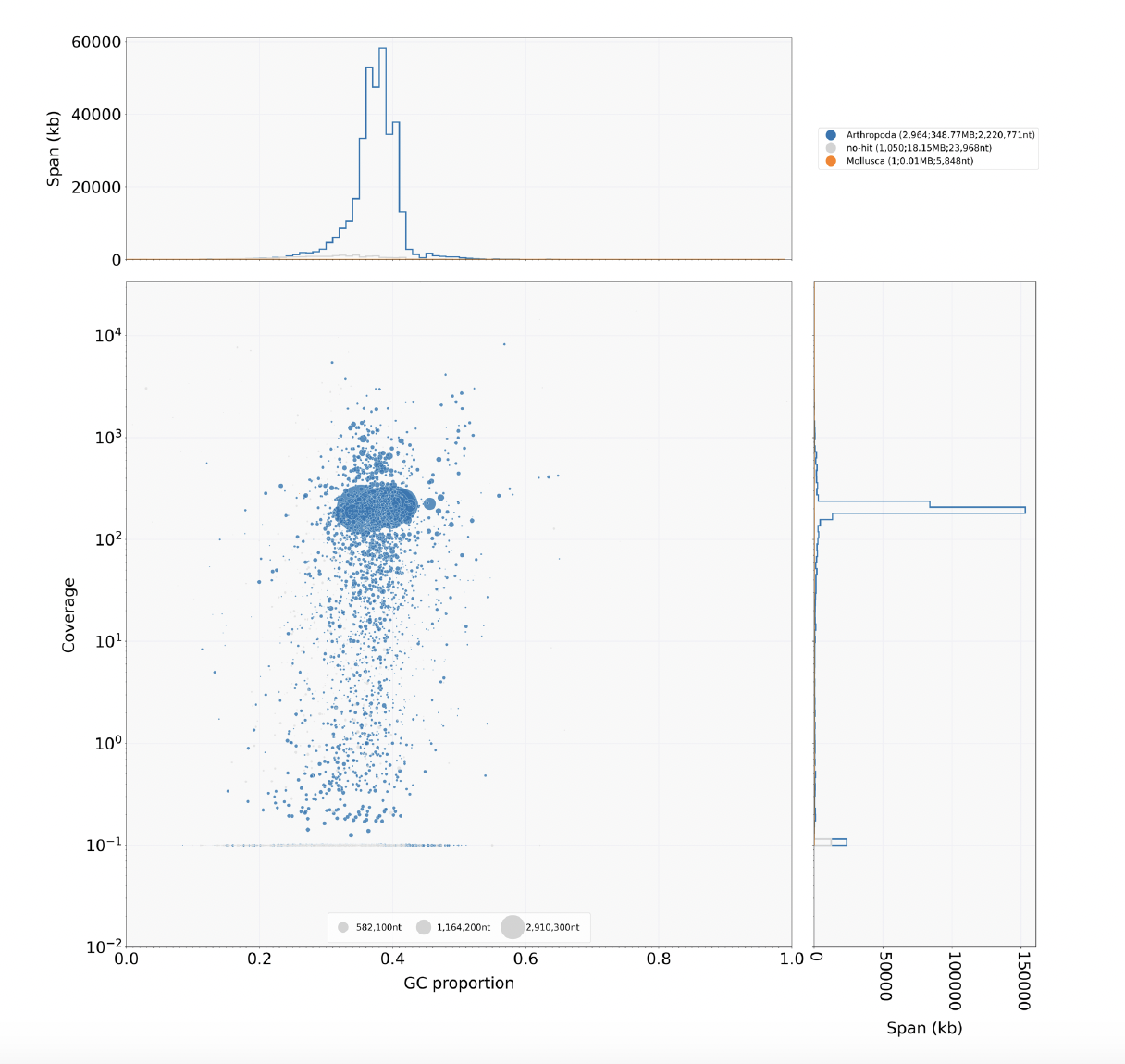
**

**Supplemental Figure S2**

Length distribution (log scale) of *B. impatiens* DNA reads from PacBio Sequencing used for the genome assembly.


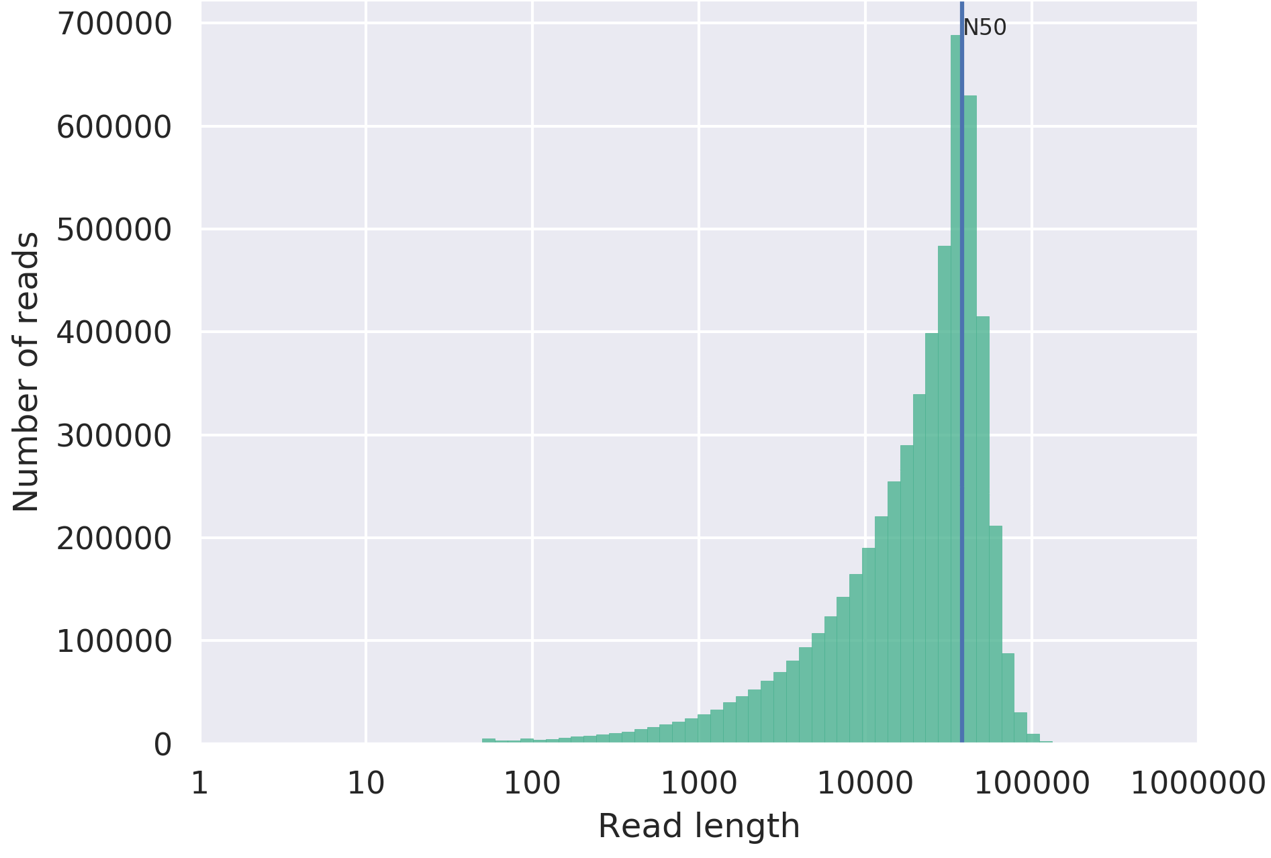


**Supplemental Figure S3**

Volcano plot of RNA-sequencing data from the brains of *B. impatiens* workers exposed to the heavy metal cocktail vs. controls, showing log_2_ fold change vs -log_10_ *P* value.

**Supplemental Figure S4**

MA plot of RNA-sequencing data from the brains of *B. impatiens* workers exposed to the heavy metal cocktail vs. controls, showing the log ratio of expression between the two groups compared to the mean average read counts (after normalization).

**Supplemental Figure S5**

Volcano plot of RNA-sequencing data from the fat bodies of *B. impatiens* workers exposed to the heavy metal cocktail vs. controls, showing log_2_ fold change vs -log_10_ *P* value.

**Supplemental Figure S6**

MA plot of RNA-sequencing data from the fat bodies of *B. impatiens* workers exposed to the heavy metal cocktail vs. controls, showing the log ratio of expression between the two groups compared to the mean average read counts (after normalization).

**Supplemental Table S1**

Assembly statistics, comparing the initial assembly and final assembly BIMP_3.0, demonstrating substantial improvements from using Juicer and manual curation. nt = nucleotides

| **Assembly** | **Initial Assembly (Dovetail)** | **Final Assembly BIMP3.0 (Juicer)** |
| --- | --- | --- |
| Number of scaffolds | 786 | 210 |
| Total size of scaffolds (nt) | 266,302,045 | 266,606,045 |
| Longest scaffold (nt) | 18,540,056 | 20,973,032 |
| Shortest scaffold (nt) | 89 | 15,040 |
| Number of scaffolds > 1K nt | 782 (99.5%) | 210 (100%) |
| Number of scaffolds > 10K nt | 589 (74.9%) | 210 (100%) |
| Number of scaffolds > 100K nt | 47 (6%) | 19 (9%) |
| Number of scaffolds > 1M nt | 19 (2.4%) | 18 (8.6%) |
| Number of scaffolds > 10M nt | 15 (1.9%) | 16 (7.6%) |
| Mean scaffold size (nt) | 338,807 | 1,269,553 |
| Median scaffold size (nt) | 20,575 | 28,615 |
| N50 scaffold length (nt) | 15,710,883 | 16,149,712 |
| L50 scaffold count | 8 | 8 |
| n90 scaffold length (nt) | 3,147,645 | 11,380,321 |
| L90 scaffold count | 19 | 16 |
| scaffold %A | 31.28 | 31.26 |
| scaffold %C | 18.73 | 18.7 |
| scaffold %G | 18.71 | 18.69 |
| scaffold %T | 31.28 | 31.22 |
| scaffold %N | 0.01 | 0.12 |
| scaffold %non-ACGTN | 0 | 0 |
| Number of scaffold non-ACGTN (nt) | 0 | 0 |
| Percentage of assembly in scaffolded contigs | 91.40% | 97.80% |
| Percentage of assembly in unscaffolded contigs | 8.60% | 2.20% |
| Average number of contigs per scaffold | 1.2 | 4.6 |
| Average length of break (> 25 Ns) between contigs in scaffold | 100 | 422 |

**Supplemental Table S2**

BUSCO statistics for the assemblies produced by the data generated in this project (initial from Dovetail HiRise and final using Juicer, designated as BIMP_3.0) and annotation.

|  | **Initial Assembly**  **(Dovetail)** | | **Final Assembly**  **BIMP_3.0 (Juicer)** | | **Annotation**  **(Braker)** | |
| --- | --- | --- | --- | --- | --- | --- |
|  | **Eukaryota (n=255)** | **Hymenoptera (n=5991)** | **Eukaryota (n=255)** | **Hymenoptera (n=5991)** | **Eukaryota (n=255)** | **Hymenoptera (n=5991)** |
| **Complete BUSCOs** | 97.60% | 95.80% | 97.60% | 96.00% | 96.1% | 93.6% |
| **Complete and single-copy BUSCOs** | 97.6% | 95.6% | 97.6% | 95.8% | 73.7% | 61.0% |
| **Complete and duplicated BUSCOs** | 0.00% | 0.20% | 0.00% | 0.20% | 22.4% | 32.6% |
| **Fragmented BUSCOs** | 1.6% | 1.5% | 1.6% | 1.3% | 2.4% | 2.9% |
| **Missing BUSCOs** | 0.80% | 2.70% | 0.80% | 2.70% | 1.5% | 3.5% |

**Supplemental Table S3**

Annotation statistics for the BIMP_3.0 annotation, CDS = coding sequence.

|  | **Gene** | **Transcript** | **CDS** |
| --- | --- | --- | --- |
| **Total bp aligned to genome** | 106,795,928 | 181,541,756 | 31,263,898 |
| **Count** | 14,011 | 21,127 | 138,723 |
| **Mean size (bp)** | 7,626 | 8,592 | 225 |
| **Median size (bp)** | 2,065 | 2,418 | 165 |
| **Min size (bp)** | 115 | 115 | 2 |
| **Max size (bp)** | 433,285 | 433,285 | 9,818 |

**Supplemental Table S4**

Repetitive and transposable elements uncovered by Repeatmasker in the BIMP_3.0 annotation.

| **Type** | **Number of** | **Length (bp)** | **Percentage (%)** |
| --- | --- | --- | --- |
| Retroelements | 19,208 | 14,934,750 | 5.6 |
| SINEs: | 0 | 0 | 0 |
| Penelope | 0 | 0 | 0 |
| LINEs: | 8,490 | 6,033,403 | 2.26 |
| CRE/SLACS | 0 | 0 | 0 |
| L2/CR1/Rex | 285 | 75,841 | 0.03 |
| R1/LOA/Jockey | 2,677 | 1,700,468 | 0.64 |
| R2/R4/NeSL | 257 | 453,758 | 0.17 |
| RTE/Bov-B | 475 | 292,825 | 0.11 |
| L1/CIN4 | 110 | 68,253 | 0.03 |
| LTR | 10,718 | 8,901,347 | 3.34 |
| BEL/Pao | 3,422 | 1,733,717 | 0.65 |
| Ty1/Copia | 881 | 732,789 | 0.27 |
| Gypsy/DIRS1 | 5,619 | 6,315,652 | 2.37 |
| Retroviral | 203 | 12,161 | 0 |
| DNA transposons | 12,728 | 3,074,277 | 1.15 |
| hobo-Activator | 1048 | 31,5203 | 0.12 |
| Tc1-IS630-Pogo | 7455 | 168,3366 | 0.63 |
| En-Spm | 0 | 0 | 0 |
| MuDR-IS905 | 0 | 0 | 0 |
| PiggyBac | 2,072 | 740,702 | 0.28 |
| Tourist/Harbinger | 109 | 2,5334 | 0.01 |
| Other (Mirage, P-element, Transib) | 0 | 0 | 0 |
| Rolling-circles | 239 | 82,433 | 0.03 |
| Unclassified | 137,962 | 33,197,315 | 12.45 |
| Total interspersed repeats | not quantified | 51,206,342 | 19.21 |
| Small RNA | 0 | 0 | 0 |
| Satellites | 0 | 0 | 0 |
| Simple repeats | 86,408 | 4,229,641 | 1.59 |
| Low complexity | 19,172 | 1,018,525 | 0.38 |

**Supplemental Table S5**

Information on samples used for Brain RNA-sequencing. ctrl= control treatment, metal = heavy metal exposed treatment

| **Well** | **Sample ID** | **Treatment** | **Filename** | **Tissue** | **Colony** | **Read length** |
| --- | --- | --- | --- | --- | --- | --- |
| A01 | A1 | ctrl | 1-A01-A1_S7_L002_R1_001.fastq | brain | A | 50 |
| A02 | A2 | ctrl | 1-A02-A2_S8_L002_R1_001.fastq | brain | A | 50 |
| A03 | A3 | ctrl | 1-A03-A3_S9_L002_R1_001.fastq | brain | A | 50 |
| A04 | A4 | ctrl | 1-A04-A4_S10_L002_R1_001.fastq | brain | A | 50 |
| A05 | A5 | ctrl | 1-A05-A5_S11_L002_R1_001.fastq | brain | A | 50 |
| A06 | A6 | ctrl | 1-A06-A6_S12_L002_R1_001.fastq | brain | A | 50 |
| A07 | A7 | ctrl | 1-A07-A7_S13_L002_R1_001.fastq | brain | A | 50 |
| A08 | A8 | ctrl | 1-A08-A8_S14_L002_R1_001.fastq | brain | A | 50 |
| A09 | A9 | ctrl | 1-A09-A9_S15_L002_R1_001.fastq | brain | A | 50 |
| A10 | A10 | ctrl | 1-A10-A10_S16_L002_R1_001.fastq | brain | A | 50 |
| A11 | A11 | ctrl | 1-A11-A11_S17_L002_R1_001.fastq | brain | A | 50 |
| A12 | A12 | ctrl | 1-A12-A12_S18_L002_R1_001.fastq | brain | A | 50 |
| B01 | A13 | ctrl | 1-B01-A13_S19_L002_R1_001.fastq | brain | A | 50 |
| B02 | A14 | ctrl | 1-B02-A14_S20_L002_R1_001.fastq | brain | A | 50 |
| B03 | A16 | ctrl | 1-B03-A16_S21_L002_R1_001.fastq | brain | A | 50 |
| B04 | A17 | ctrl | 1-B04-A17_S22_L002_R1_001.fastq | brain | A | 50 |
| B05 | A18 | ctrl | 1-B05-A18_S23_L002_R1_001.fastq | brain | A | 50 |
| B06 | A19 | ctrl | 1-B06-A19_S24_L002_R1_001.fastq | brain | A | 50 |
| B07 | B2 | metal | 1-B07-B2_S25_L002_R1_001.fastq | brain | B | 50 |
| B08 | B3 | metal | 1-B08-B3_S26_L002_R1_001.fastq | brain | B | 50 |
| B09 | B4 | metal | 1-B09-B4_S27_L002_R1_001.fastq | brain | B | 50 |
| B10 | B5 | metal | 1-B10-B5_S28_L002_R1_001.fastq | brain | B | 50 |
| B11 | B6 | metal | 1-B11-B6_S29_L002_R1_001.fastq | brain | B | 50 |
| B12 | B7 | metal | 1-B12-B7_S30_L002_R1_001.fastq | brain | B | 50 |
| C01 | B8 | metal | 1-C01-B8_S31_L002_R1_001.fastq | brain | B | 50 |
| C02 | B10 | metal | 1-C02-B10_S32_L002_R1_001.fastq | brain | B | 50 |
| C03 | C1 | ctrl | 1-C03-C1_S33_L002_R1_001.fastq | brain | C | 50 |
| C04 | C2 | ctrl | 1-C04-C2_S34_L002_R1_001.fastq | brain | C | 50 |
| C05 | C3 | ctrl | 1-C05-C3_S35_L002_R1_001.fastq | brain | C | 50 |
| C06 | C4 | ctrl | 1-C06-C4_S36_L002_R1_001.fastq | brain | C | 50 |
| C07 | C6 | ctrl | 1-C07-C6_S37_L002_R1_001.fastq | brain | C | 50 |
| C08 | C7 | ctrl | 1-C08-C7_S38_L002_R1_001.fastq | brain | C | 50 |
| C09 | C8 | ctrl | 1-C09-C8_S39_L002_R1_001.fastq | brain | C | 50 |
| C10 | C9 | ctrl | 1-C10-C9_S40_L002_R1_001.fastq | brain | C | 50 |
| C11 | D1 | metal | 1-C11-D1_S41_L002_R1_001.fastq | brain | D | 50 |
| C12 | D2 | metal | 1-C12-D2_S42_L002_R1_001.fastq | brain | D | 50 |
| D01 | D5 | metal | 1-D01-D5_S43_L002_R1_001.fastq | brain | D | 50 |
| D02 | D6 | metal | 1-D02-D6_S44_L002_R1_001.fastq | brain | D | 50 |
| D03 | D7 | metal | 1-D03-D7_S45_L002_R1_001.fastq | brain | D | 50 |
| D04 | D8 | metal | 1-D04-D8_S46_L002_R1_001.fastq | brain | D | 50 |
| D05 | D9 | metal | 1-D05-D9_S47_L002_R1_001.fastq | brain | D | 50 |
| D06 | D10 | metal | 1-D06-D10_S48_L002_R1_001.fastq | brain | D | 50 |
| D07 | D11 | metal | 1-D07-D11_S49_L002_R1_001.fastq | brain | D | 50 |
| D08 | E1 | metal | 1-D08-E1_S50_L002_R1_001.fastq | brain | E | 50 |
| D09 | E2 | metal | 1-D09-E2_S51_L002_R1_001.fastq | brain | E | 50 |
| D10 | E5 | metal | 1-D10-E5_S52_L002_R1_001.fastq | brain | E | 50 |
| D11 | E7 | metal | 1-D11-E7_S53_L002_R1_001.fastq | brain | E | 50 |
| D12 | E8 | metal | 1-D12-E8_S54_L002_R1_001.fastq | brain | E | 50 |
| E01 | E9 | metal | 1-E01-E9_S55_L002_R1_001.fastq | brain | E | 50 |
| E02 | E10 | metal | 1-E02-E10_S56_L002_R1_001.fastq | brain | E | 50 |
| E03 | F1 | metal | 1-E03-F1_S57_L002_R1_001.fastq | brain | F | 50 |
| E04 | F2 | metal | 1-E04-F2_S58_L002_R1_001.fastq | brain | F | 50 |
| E05 | F3 | metal | 1-E05-F3_S59_L002_R1_001.fastq | brain | F | 50 |
| E06 | F4 | metal | 1-E06-F4_S60_L002_R1_001.fastq | brain | F | 50 |
| E07 | F5 | metal | 1-E07-F5_S61_L002_R1_001.fastq | brain | F | 50 |
| E08 | F6 | metal | 1-E08-F6_S62_L002_R1_001.fastq | brain | F | 50 |
| E09 | F7 | metal | 1-E09-F7_S63_L002_R1_001.fastq | brain | F | 50 |
| E10 | F8 | metal | 1-E10-F8_S64_L002_R1_001.fastq | brain | F | 50 |
| E11 | F9 | metal | 1-E11-F9_S65_L002_R1_001.fastq | brain | F | 50 |
| E12 | F10 | metal | 1-E12-F10_S66_L002_R1_001.fastq | brain | F | 50 |

**Supplemental Table S6**

Information on samples used for Fat body RNA-sequencing. ctrl= control treatment, metal = heavy metal exposed treatment

| **Well** | **Sample ID** | **Treatment** | **Filename** | **Tissue** | **Colony** | **Read length** |
| --- | --- | --- | --- | --- | --- | --- |
| A01 | A3 | ctrl | 1-A01-A3_S1_L002_R1_001.fastq | fat body | A | 100 |
| A02 | E4 | metal | 1-A02-E4_S2_L002_R1_001.fastq | fat body | E | 100 |
| A03 | E5 | metal | 1-A03-E5_S3_L002_R1_001.fastq | fat body | E | 100 |
| A04 | A15 | ctrl | 1-A04-A15_S4_L002_R1_001.fastq | fat body | A | 100 |
| A05 | F8 | metal | 1-A05-F8_S5_L002_R1_001.fastq | fat body | F | 100 |
| A06 | D2 | metal | 1-A06-D2_S6_L002_R1_001.fastq | fat body | D | 100 |
| A07 | D1 | metal | 1-A07-D1_S7_L002_R1_001.fastq | fat body | D | 100 |
| A08 | A2 | ctrl | 1-A08-A2_S8_L002_R1_001.fastq | fat body | A | 100 |
| A09 | F9 | metal | 1-A09-F9_S9_L002_R1_001.fastq | fat body | F | 100 |
| A10 | B9 | metal | 1-A10-B9_S10_L002_R1_001.fastq | fat body | B | 100 |
| A11 | B7 | metal | 1-A11-B7_S11_L002_R1_001.fastq | fat body | B | 100 |
| A12 | A13 | ctrl | 1-A12-A13_S12_L002_R1_001.fastq | fat body | A | 100 |
| B01 | A14 | ctrl | 1-B01-A14_S13_L002_R1_001.fastq | fat body | A | 100 |
| B02 | A1 | ctrl | 1-B02-A1_S14_L002_R1_001.fastq | fat body | A | 100 |
| B03 | F10 | metal | 1-B03-F10_S15_L002_R1_001.fastq | fat body | F | 100 |
| B04 | B8 | metal | 1-B04-B8_S16_L002_R1_001.fastq | fat body | B | 100 |
| B05 | B11 | metal | 1-B05-B11_S17_L002_R1_001.fastq | fat body | B | 100 |
| B06 | F10 | metal | 1-B06-F10_S18_L002_R1_001.fastq | fat body | F | 100 |
| B07 | C1 | ctrl | 1-B07-C1_S19_L002_R1_001.fastq | fat body | C | 100 |
| B08 | D5 | metal | 1-B08-D5_S20_L002_R1_001.fastq | fat body | D | 100 |
| B09 | C3 | ctrl | 1-B09-C3_S21_L002_R1_001.fastq | fat body | C | 100 |
| B10 | A4 | ctrl | 1-B10-A4_S22_L002_R1_001.fastq | fat body | A | 100 |
| B11 | A5 | ctrl | 1-B11-A5_S23_L002_R1_001.fastq | fat body | A | 100 |
| B12 | C2 | ctrl | 1-B12-C2_S24_L002_R1_001.fastq | fat body | C | 100 |
| C01 | E7 | metal | 1-C01-E7_S25_L002_R1_001.fastq | fat body | E | 100 |
| C02 | D4 | metal | 1-C02-D4_S26_L002_R1_001.fastq | fat body | D | 100 |
| C03 | A17 | ctrl | 1-C03-A17_S27_L002_R1_001.fastq | fat body | A | 100 |
| C04 | A6 | ctrl | 1-C04-A6_S28_L002_R1_001.fastq | fat body | A | 100 |
| C05 | A16 | ctrl | 1-C05-A16_S29_L002_R1_001.fastq | fat body | A | 100 |
| C06 | D3 | metal | 1-C06-D3_S30_L002_R1_001.fastq | fat body | D | 100 |
| C07 | A18 | ctrl | 1-C07-A18_S31_L002_R1_001.fastq | fat body | A | 100 |
| C08 | E8 | metal | 1-C08-E8_S32_L002_R1_001.fastq | fat body | E | 100 |
| C09 | B10 | metal | 1-C09-B10_S33_L002_R1_001.fastq | fat body | B | 100 |
| C10 | E6 | metal | 1-C10-E6_S34_L002_R1_001.fastq | fat body | E | 100 |
| C11 | C6 | ctrl | 1-C11-C6_S35_L002_R1_001.fastq | fat body | C | 100 |
| C12 | D9 | metal | 1-C12-D9_S36_L002_R1_001.fastq | fat body | D | 100 |
| D01 | F7 | metal | 1-D01-F7_S37_L002_R1_001.fastq | fat body | F | 100 |
| D02 | D7 | metal | 1-D02-D7_S38_L002_R1_001.fastq | fat body | D | 100 |
| D03 | C8 | ctrl | 1-D03-C8_S39_L002_R1_001.fastq | fat body | C | 100 |
| D04 | B6 | metal | 1-D04-B6_S40_L002_R1_001.fastq | fat body | B | 100 |
| D05 | A12 | ctrl | 1-D05-A12_S41_L002_R1_001.fastq | fat body | A | 100 |
| D06 | E1 | metal | 1-D06-E1_S42_L002_R1_001.fastq | fat body | E | 100 |
| D07 | A9 | ctrl | 1-D07-A9_S43_L002_R1_001.fastq | fat body | A | 100 |
| D08 | C9 | ctrl | 1-D08-C9_S44_L002_R1_001.fastq | fat body | C | 100 |
| D09 | B3 | metal | 1-D09-B3_S45_L002_R1_001.fastq | fat body | B | 100 |
| D10 | A11 | ctrl | 1-D10-A11_S46_L002_R1_001.fastq | fat body | A | 100 |
| D11 | F4 | metal | 1-D11-F4_S47_L002_R1_001.fastq | fat body | F | 100 |
| D12 | E2 | metal | 1-D12-E2_S48_L002_R1_001.fastq | fat body | E | 100 |
| E01 | E3 | metal | 1-E01-E3_S49_L002_R1_001.fastq | fat body | E | 100 |
| E02 | B4 | metal | 1-E02-B4_S50_L002_R1_001.fastq | fat body | B | 100 |
| E03 | C7 | ctrl | 1-E03-C7_S51_L002_R1_001.fastq | fat body | C | 100 |
| E04 | A8 | ctrl | 1-E04-A8_S52_L002_R1_001.fastq | fat body | A | 100 |
| E05 | B1 | metal | 1-E05-B1_S53_L002_R1_001.fastq | fat body | B | 100 |
| E06 | E9 | metal | 1-E06-E9_S54_L002_R1_001.fastq | fat body | F | 100 |
| E07 | F5 | metal | 1-E07-F5_S55_L002_R1_001.fastq | fat body | F | 100 |
| E08 | F6 | metal | 1-E08-F6_S56_L002_R1_001.fastq | fat body | F | 100 |
| E09 | B5 | metal | 1-E09-B5_S57_L002_R1_001.fastq | fat body | B | 100 |
| E10 | A10 | ctrl | 1-E10-A10_S58_L002_R1_001.fastq | fat body | A | 100 |
| E11 | C4 | ctrl | 1-E11-C4_S59_L002_R1_001.fastq | fat body | C | 100 |
| E12 | E10 | metal | 1-E12-E10_S60_L002_R1_001.fastq | fat body | E | 100 |
| F01 | F3 | metal | 1-F01-F3_S61_L002_R1_001.fastq | fat body | F | 100 |
| F02 | A7 | ctrl | 1-F02-A7_S62_L002_R1_001.fastq | fat body | A | 100 |
| F03 | F2 | metal | 1-F03-F2_S63_L002_R1_001.fastq | fat body | F | 100 |
| F04 | D8 | metal | 1-F04-D8_S64_L002_R1_001.fastq | fat body | D | 100 |
| F05 | C5 | ctrl | 1-F05-C5_S65_L002_R1_001.fastq | fat body | C | 100 |
| F06 | B2 | metal | 1-F06-B2_S66_L002_R1_001.fastq | fat body | B | 100 |
| F07 | D6 | metal | 1-F07-D6_S67_L002_R1_001.fastq | fat body | D | 100 |

**Supplemental Table S7**

Summary of RNA-seq differential expression analysis, comparing control vs metal exposed brains and fat bodies. Results for two different significance thresholds are shown, adjusted p-value cutoff < 0.1 or <0.05.

| **Tissue** | **Brain (p<0.1)** | **Fat Body (p<0.1)** | **Brain (p<0.05)** | **Fat Body (p<0.05)** |
| --- | --- | --- | --- | --- |
| Total transcripts with nonzero read count | 13,113 | 13,502 | 13114 | 13502 |
| Differentially expressed transcript count (% of total nonzero) | 999 (7.6%) | 57 (0.4%) | 603 (4.6%) | 34 (0.23%) |
| Upregulated transcript count | 542 | 36 | 319 | 23 |
| Downregulated transcript count | 457 | 21 | 284 | 11 |
| Outlier transcript count | 0 | 0 | 0 | 0 |
| Low reads (<1 mean reads) transcript count | 6,859 | 4,447 | 6859 | 4708 |
| Total transcripts analyzed | 20,971 | 18,006 | 19973 | 18210 |

**Supplemental Datasets**

**Supplemental Dataset 1**

Single file containing a conversion list of new BIMP_3.0 annotation to previous annotation BIMP_2.2, functional annotations for the annotated genes from the BIMP_3.0 annotation, RNA-seq statistical results from DESeq2 for both fat body and brain tissues

<https://github.com/ISUgenomics/2024_Toth_Bimpatiens/blob/main/Supplementary_Data_File_All.xlsx>

**Supplemental Dataset 2**

Conversion list for annotations from BIMP_3.0 compared to the earlier annotation NCBI Release 103, associated with BIMP_2.2 genome.

<https://github.com/ISUgenomics/2024_Toth_Bimpatiens/blob/main/Notebook_Masonbrink/05_AlignNCBIAnnotation.md>

**Supplemental Dataset 3**

Quality statistics on brain RNA-sequencing data

<https://github.com/ISUgenomics/2024_Toth_Bimpatiens/blob/main/Notebook_Chris/data/multiqc/multiqc_brain_report.html.gz>

**Supplemental Dataset 4**

Quality statistics on fat body RNA-sequencing data

<https://github.com/ISUgenomics/2024_Toth_Bimpatiens/blob/main/Notebook_Chris/data/multiqc/multiqc_fatbody_report.html.gz>
